# p38γ and p38δ MAP kinases in inflammation

**DOI:** 10.1042/BST20250382

**Published:** 2026-10-06

**Authors:** Ester Díaz-Mora, Ana Risco, Ana Cuenda

**Affiliations:** Department of Immunology and Oncology, Centro Nacional de Biotecnología (CNB)/CSIC, Madrid, Spain

**Keywords:** cytokine, innate immunity, macrophage, MAP kinase, p38γ, p38δ

## Abstract

The p38γ and p38δ mitogen-activated protein kinases (MAPKs) are members of the p38 family that have emerged as important regulators of inflammatory signaling. Initially considered minor or alternative isoforms, accumulating evidence has revealed distinct and overlapping functions for p38γ and p38δ in innate immunity, tissue homeostasis, inflammatory disease, and cancer. These kinases regulate immune responses through diverse mechanisms controlling cytokine production, gene expression, immune-cell activation, and migration. In addition, recent studies suggest that some functions of p38γ and p38δ may occur independently of their catalytic activity, highlighting the complexity of their biological roles. The present review summarizes current knowledge of the molecular features, substrates, immune cell functions, and disease associations of p38γ and p38δ and discusses their potential as therapeutic targets while addressing the challenges associated with their pharmacological modulation.

## Introduction

The p38 mitogen-activated protein kinase (MAPK) family comprises four isoforms: p38α (*Mapk14*), p38β (*Mapk11*), p38γ (*Mapk12*; also known as ERK6 or SAPK3), and p38δ (*Mapk13*; also known as SAPK4) [1,2]. These isoforms share approximately 60% amino acid identity but differ in their tissue distribution, substrate preference, activation mechanisms, and sensitivity to pharmacological inhibitors [1,2]. Comparative genomic and functional studies indicate that the p38MAPK pathway was already present in unicellular holozoans before the emergence of multicellular animals, where it likely functioned as a conserved stress-responsive module involved in environmental adaptation, particularly osmotic stress responses [1–4]. During vertebrate evolution, successive gene duplication events generated the four p38 paralogues, with p38γ and p38δ emerging as closely related isoforms that subsequently acquired specialized, but not necessarily exclusive, functions [3].

The p38MAPK field has historically been dominated by studies of p38α, whose identification as a target of pyridinyl-imidazole compounds that suppress lipopolysaccharide (LPS)-induced cytokine synthesis established it as the prototypical inflammatory p38MAPK. Consequently, most studies referring generically to ‘p38’ have focused on this isoform [1,5]. The selective inhibition of p38α and p38β (p38α/p38β) by SB203580, while sparing p38γ and p38δ (p38γ/p38δ), further contributed to the classification of the latter as ‘alternative’ p38MAPKs and limited their investigation for many years [2,6]. This terminology, however, reflects a historical distinction and should not be taken to imply that p38γ/p38δ are secondary or redundant members of the family. Evidence from genetic and functional studies indicates that these isoforms make important and, in some contexts, non-redundant contributions that are not fully compensated for by p38α or p38β [7,8]. At the same time, because several p38 isoforms are co-expressed in many cell types, their functions may be distinct, overlapping, or compensatory depending on their relative abundance, activation kinetics, subcellular localization, and access to substrates, binding partners, and scaffolding proteins [7]. Phenotypes resulting from the deletion or inhibition of an individual isoform should therefore be interpreted in the context of potential contributions from other p38 family members.

The limited clinical success of p38α/p38β inhibitors in chronic inflammatory diseases, despite their effective suppression of cytokine production in early studies, together with adverse effects such as hepatotoxicity and the development of tachyphylaxis, prompted a re-evaluation of the roles of the other p38 isoforms [9]. Studies based largely on isoform-specific and combined knockout mouse models have since identified p38γ and p38δ as active components of inflammatory signaling networks and revealed their context-dependent contribution to physiological and pathological processes [2,10].

p38γ and p38δ regulate inflammatory responses through canonical phosphorylation-dependent pathways as well as non-canonical mechanisms, including kinase-independent effects on the stability and organization of signaling complexes [2,10,11]. However, non-catalytic functions are not exclusive to p38γ and p38δ and have also been described for other p38 family members [4,12]. The key unresolved question is how cellular and stimulus-dependent factors shape the individual and overlapping functions of p38γ and p38δ within the broader p38 signaling network.

The present review summarizes current knowledge of p38γ and p38δ in inflammatory signaling, focusing on their unique molecular features, immune functions, roles in inflammatory diseases, and the challenges associated with their therapeutic targeting.

## Molecular features of p38γ and p38δ

Although p38γ and p38δ share the conserved structural organization and activation mechanism characteristic of the p38MAPK family, they also possess distinctive molecular features that confer unique signaling properties. Like all p38MAPKs, both isoforms contain the conserved activation-loop TGY motif, whose dual phosphorylation by the MAP2Ks MKK3 and MKK6 is required for kinase activation [4,5,13]. Although MKK4 has been shown to phosphorylate all p38 isoforms *in vitro*, genetic studies using MKK-deficient cells have demonstrated that activation of p38γ and p38δ in physiological settings is primarily mediated by MKK3 and MKK6 [13]. Importantly, activation of these kinases is both isoform- and stimulus-dependent. Whereas stress-induced activation of p38γ requires both MKK3 and MKK6, MKK6 predominates in response to TNFα. In contrast, p38δ activation by cellular stresses and TNFα depends predominantly on MKK3 [13]. Upstream of these MAP2Ks, MAP3Ks such as transforming growth factor-β-activated kinase 1 (MAP3K7) integrate signals downstream of Toll-like receptors (TLRs), the IL-1 receptor, and C-type lectin receptors, including Dectin-1, thereby linking inflammatory stimuli to p38γ/p38δ activation [2,14].

Despite sharing 70% sequence similarity [15], p38γ and p38δ exhibit important structural differences that contribute to their functional specialization. A unique feature of p38γ is the acquisition of a C-terminal –KETXL PDZ-binding motif, absent from all other p38 isoforms, which mediates interactions with PDZ domain-containing scaffold proteins such as α-Syntrophin, protein tyrosine phosphatase H1, and hDlg/synapse-associated protein 97 (DLG1) [2]. Through these interactions, p38γ assembles isoform-specific signaling complexes and can regulate cellular processes beyond its canonical kinase activity. Another important distinguishing feature of p38γ and p38δ is the composition of the ATP-binding pocket gatekeeper region, which renders both isoforms insensitive to pyridinyl-imidazole inhibitors such as SB203580 [1,5]. By contrast, the allosteric inhibitor BIRB796 (doramapimod), which stabilizes an inactive DFG-out conformation, inhibits all four p38 isoforms with different potencies and has therefore become an important pharmacological tool for investigating p38γ/p38δ-dependent signaling events that are insensitive to SB203580 [2,16–18].

## Functions of p38γ and p38δ in the immune response

The functions of p38γ and p38δ in innate immune cells are mediated through a relatively restricted set of substrates and signaling modules that distinguish these kinases from the canonical p38α pathway. Rather than acting exclusively as catalytic enzymes, p38γ and p38δ also function as regulators of signaling architecture, influencing the stability, assembly, and activity of multiprotein complexes that control inflammatory responses. Through both kinase-dependent and kinase-independent mechanisms, these kinases regulate cytokine production, gene expression, protein translation, antimicrobial responses, and leukocyte migration [2,11,19] (Figure 1).

**Figure 1:**
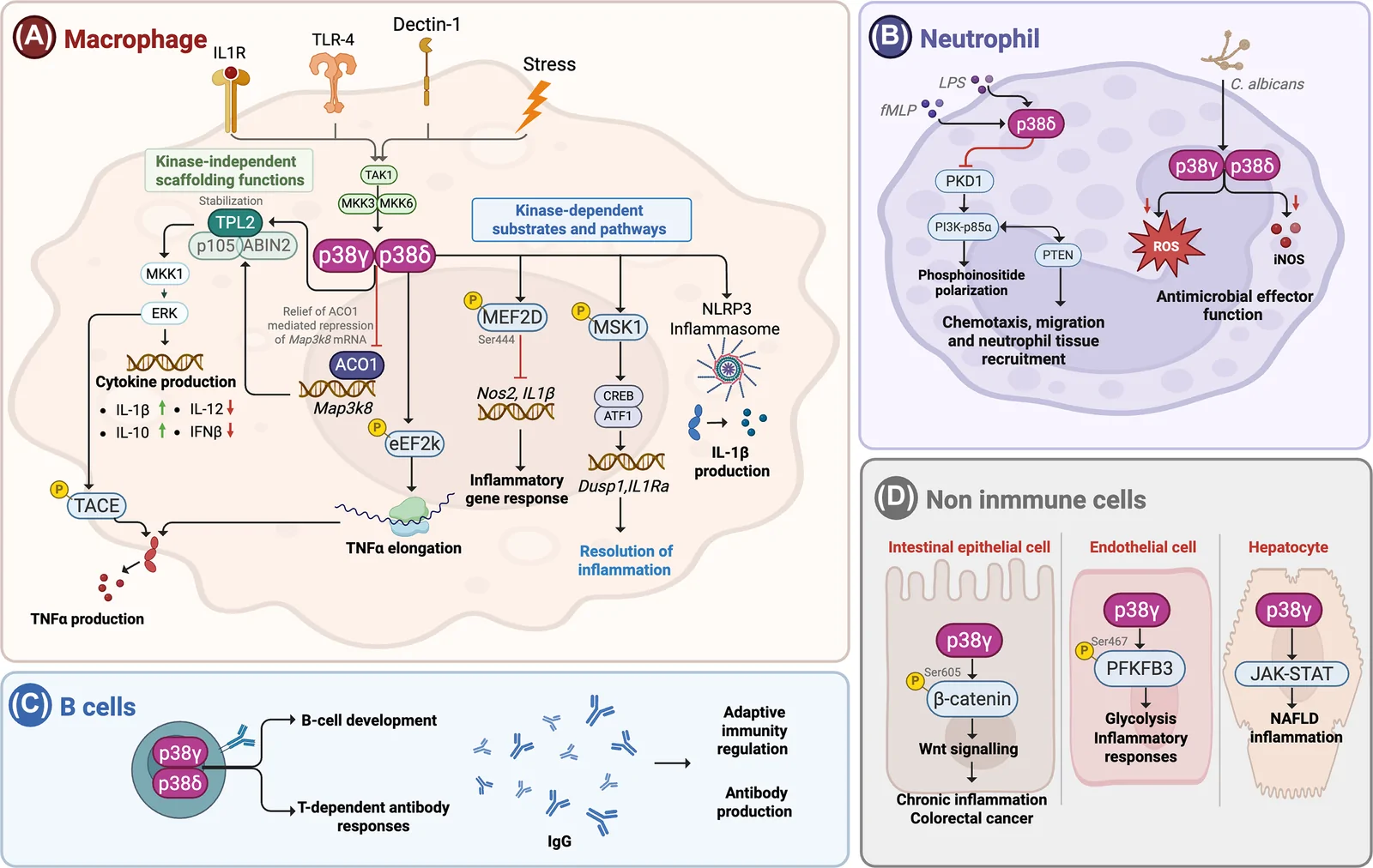
Roles of p38γ and p38δ in the regulation of inflammatory responses in immune and non-immune cells p38γ and p38δ exert both kinase-dependent and kinase-independent functions that modulate inflammatory and immune responses. In macrophages (**A**), these kinases regulate cytokine production, TNFα expression, inflammatory gene transcription, inflammasome activation, and the resolution of inflammation through distinct signaling pathways and scaffold functions. In neutrophils (**B**), p38γ and p38δ control chemotaxis, migration, tissue recruitment, and antimicrobial effector functions, including reactive oxygen species (ROS) and inducible nitric oxide synthase (iNOS) production. In B cells (**C**), p38γ and p38δ contribute to B-cell development and T cell-dependent antibody responses. In non-immune cells (**D**), p38γ regulates diverse pathological processes, including Wnt/β-catenin signaling in intestinal epithelial cells, glycolytic and inflammatory responses in endothelial cells, and JAK-STAT (Janus kinase–signal transducer and activator of transcription) signaling associated with hepatic inflammation. Overall, p38γ and p38δ play critical roles in coordinating innate immunity, adaptive immunity, and inflammatory disease processes. Created with BioRender.com (https://BioRender.com/94ka8mx).

A central component of p38γ/p38δ signaling in macrophages and dendritic cells is tumor progression locus 2 (TPL2/MAP3K8), a key activator of the ERK1/2 pathway downstream of TLRs and other innate immune receptors [11,14,20]. TPL2 normally exists in a cytoplasmic complex with ABIN2 (A20-binding inhibitor of NF-κB 2) and the NF-κB1 precursor p105, from which it mediates MKK1/2 and ERK1/2 activation following receptor stimulation [11,21]. Genetic deletion of p38γ and p38δ markedly reduces TPL2 protein levels, resulting in impaired ERK1/2 activation and altered cytokine production, characterized by reduced TNFα, IL-1β, and IL-10 but increased IL-12 and interferon beta expression [20]. Mechanistically, p38γ/p38δ regulate TPL2 through two complementary post-transcriptional mechanisms: stabilization of the TPL2-ABIN2-p105 complex and relief of translational repression mediated by aconitase-1 binding to the 3′ untranslated region of Map3k8 mRNA [11]. Importantly, both mechanisms occur independently of kinase activity, demonstrating that p38γ/p38δ can function as structural components of inflammatory signaling complexes rather than solely as protein kinases [11].

In addition to controlling TPL2, p38γ and p38δ regulate inflammatory gene expression through direct phosphorylation of downstream substrates. Studies using p38γ/δ^KIKO^ (p38γ^D171A/D171A^/p38δ^−/−^) mice that express a kinase-inactive p38γ mutant while lacking p38δ, which preserves TPL2 expression, identified MEF2D (myocyte enhancer factor-2D) as a direct substrate of p38γ/p38δ in innate immune cells [22]. Phosphorylation of MEF2D on Ser444 (Ser437 in mice) suppresses its transcriptional activity, whereas non-phosphorylatable mutants enhance expression of pro-inflammatory genes, including Nos2 and Il1b [22]. Consistent with these findings, p38γ/δ^KIKO^ mice display attenuated inflammatory responses and reduced susceptibility to LPS-induced septic shock and *Candida albicans* infection [22]. MEF2D therefore represents a direct link between p38γ/p38δ kinase activity and inflammatory gene transcription.

Another signaling pathway controlled by these kinases involves p38δ-dependent activation of mitogen- and stress-activated protein kinase 1 (MSK1) in macrophages following TLR stimulation. Through the MSK1-CREB/ATF1 axis, p38δ regulates the expression of genes involved in the resolution and control of inflammatory responses, including dual-specificity phosphatase 1 and interleukin-1 receptor antagonist, revealing an additional mechanism through which alternative p38 MAPKs modulate innate immunity [23].

Besides regulating gene transcription, p38γ/p38δ also control cytokine synthesis at the translational level. One of the earliest substrates identified for p38δ was eukaryotic elongation factor 2 kinase (eEF2K), which is phosphorylated on Ser359, resulting in inhibition of eEF2K activity and activation of eEF2-dependent translational elongation [24]. During endotoxin responses, activation of the MKK3/6-p38γ/p38δ pathway promotes TNFα production through enhanced translational elongation rather than through changes in gene transcription or mRNA stability [25]. Consistent with this mechanism, mice lacking p38γ/p38δ produce less TNFα and are protected against LPS-induced hepatitis, whereas inhibition of p38α/β exacerbates liver injury, highlighting important functional differences among p38 family members [25].

Another important downstream effect of these kinases is the role of p38δ in atherosclerotic plaques, where it regulates NLRP3 inflammasome activation in human macrophages. p38δ is activated by ATP and cholesterol crystals and is required for NLRP3-dependent IL-1β secretion through a mechanism that remains incompletely understood [26].

p38γ and p38δ also regulate immune-cell migration and tissue recruitment. Neutrophils express particularly high levels of p38δ, and one of the best-characterized substrates mediating its function is protein kinase D1 (PKD1) [27]. In neutrophils, p38δ inhibits PKD1 activity, thereby regulating PKD1-dependent phosphorylation of the PI3K regulatory subunit p85α and controlling PTEN (phosphatase and tensin homolog) activity, phosphoinositide polarization, and directional migration [27]. Through the PKD1-PTEN pathway, p38δ coordinates leukocyte polarity and chemotaxis, contributing to neutrophil recruitment during inflammatory responses [27]. Genetic disruption of this pathway impairs neutrophil migration and reduces acute inflammatory lung injury, demonstrating that p38δ regulates not only cytokine production but also the cellular trafficking events that shape inflammatory responses [27].

In addition to cytokine production and cell migration, p38γ/p38δ regulate antimicrobial effector functions in phagocytic cells. Studies using *C. albicans* infection models revealed that macrophages and neutrophils lacking both kinases display enhanced fungicidal activity despite exhibiting reduced inflammatory cytokine production [18]. Increased fungal killing is associated with elevated production of ROS and iNOS, resulting in improved clearance of *C. albicans* by p38γ/p38δ-deficient phagocytes [18].

Beyond classical innate immune responses, several additional p38γ substrates have been associated with inflammation-related processes in non-immune cells. In intestinal epithelial cells, p38γ phosphorylates β-catenin at Ser605, enhancing its stability and promoting Wnt signaling, thereby contributing to chronic intestinal inflammation and colitis-associated colorectal cancer [28,29]. Likewise, p38γ regulates PFKFB3, linking inflammatory signaling to glycolytic reprogramming. Direct phosphorylation of PFKFB3 at Ser467 has been reported in Kirsten rat sarcoma viral oncogene homolog-driven pancreatic cancer models [30], whereas more recent studies in liver sinusoidal endothelial cells identified PFKFB3 as a downstream mediator of p38γ-dependent glycolysis and inflammatory responses during alcoholic liver disease [31]. Furthermore, p38γ expression is increased in hepatocytes during metabolic dysfunction-associated steatotic liver disease (MASLD; formerly NAFLD), where its silencing alleviates hepatic steatosis, inflammation, and liver injury through mechanisms involving JAK-STAT signaling, further supporting a role for p38γ in metabolic inflammation [32].

Other substrates may provide additional links between p38γ and p38δ signaling and inflammation. p62/SQSTM1 functions as a signaling adaptor linking autophagy, NF-κB activation, and innate immune responses, thereby contributing to chronic inflammation and metabolic disorders [33–35]. Similarly, coactivator-associated arginine methyltransferase 1 (CARM1) has emerged as a potential connection between p38γ signaling and immune regulation. In conventional type 1 dendritic cells, CARM1 limits antigen presentation and T-cell priming [36], whereas in 10T1/2 mesenchymal fibroblast-like cells, p38γ-dependent phosphorylation of CARM1 promotes ROS production and cellular senescence [37]. Although a direct role for the p38γ-CARM1 axis in immune cells remains to be established, these observations suggest that p38γ may modulate inflammatory responses through epigenetic, metabolic, and stress-associated pathways.

p38γ/p38δ also contribute to adaptive immune responses. Genetic studies have shown that these kinases regulate B-cell development and the T-dependent antibody response, with combined deficiency resulting in altered humoral immunity [38]. These findings indicate that p38γ/p38δ influence both innate and adaptive immunity and suggest that inhibition of these kinases may have broader immunological consequences than anticipated.

Collectively, the available evidence indicates that p38γ and p38δ regulate innate immune responses through multiple complementary mechanisms (Figure 1). These include kinase-independent scaffolding functions that control TPL2 stability and signaling, direct phosphorylation of substrates involved in transcription (MEF2D and MSK1), translation (eEF2K), cell migration (PKD1), inflammasome activation, and antimicrobial responses, as well as emerging roles in immunometabolism and inflammation-associated tumorigenesis mediated by β-catenin, PFKFB3, p62, and CARM1. This functional diversity explains why complete genetic ablation of p38γ/p38δ often produces broader biological effects than inhibition of kinase activity alone and highlights the need to consider both catalytic and non-catalytic functions when developing therapeutic strategies targeting these kinases.

## p38γ/p38δ in inflammatory disease models

Beyond mechanistic studies, accumulating evidence links p38γ and p38δ to a broad spectrum of inflammatory and inflammation-associated diseases [2]. In human tissues and patient-derived samples, altered expression or activity of one or both kinases has been associated with disease state, inflammatory burden, progression, or clinical outcome [39–42]. These observations, derived from transcriptomic and RNA-sequencing datasets, histopathological analysis, and patient samples, establish clinically relevant associations but do not demonstrate that p38γ or p38δ initiate disease. Stronger evidence for a functional contribution has been obtained from RNA interference, gene silencing, and constitutive and conditional knockout and kinase-inactive mouse models [20,22,25,27]. Across diverse experimental models of systemic, infectious, autoimmune, metabolic, and tissue-specific inflammation, disruption of p38γ and/or p38δ signaling frequently attenuates pathological inflammation and tissue damage (Table 1). The context-dependent and sometimes divergent phenotypes observed across disease models probably reflect differences in the cellular sources and functions of p38γ and p38δ, as well as potential compensation and signaling redistribution among co-expressed p38 isoforms. Together, these findings support p38γ and p38δ not only as biomarkers associated with inflammatory disease activity but also as context-dependent modulators of disease development or severity and potential therapeutic targets [2,40]. In particular, loss-of-function studies provide strong evidence that p38γ and p38δ are functionally required in specific pathological settings, although complementary gain-of-function approaches are needed to determine whether their activation is sufficient to promote disease. The interpretation of constitutive or whole-body knockout models should nevertheless consider potential developmental compensation and the contribution of both cell-intrinsic and systemic effects. Inducible, cell-type-specific models will be important to define more precisely the causal contribution of these kinases in specific pathological contexts. The principal disease models and pathological conditions in which p38γ and/or p38δ have been implicated are summarized in Table 1.

**Table 1:** Involvement of p38γ and p38δ in inflammatory diseases and their therapeutic potential

Disease	p38 isoform(s)	Evidence	Key findings	Ref.
Atherosclerosis	p38δ	Human coronary artery RNA-seq; human atherosclerotic plaques; immunohistochemistry; primary human macrophages stimulated with cholesterol crystals or ATP	p38δ expression is increased during atherogenesis and colocalizes with foam cells. p38δ is activated by cholesterol crystals and ATP and is required for NLRP3 inflammasome activation and IL-1β secretion.	[26]
Endotoxic shock/Sepsis	p38γ/p38δ	p38γ/δ^−/−^, p38γ^−/−^, p38δ^−/−^, p38γ/δ^KIKO^ mice; LPS challenge; macrophage signaling studies	Genetic inactivation reduces circulating TNFα and IL-1β, impairs TLR4-TPL2-ERK1/2 signaling, and protects against LPS-induced septic shock.	[20,22]
Acute lung injury (ALI)	p38δ	p38δ^−/−^ mice; LPS-induced lung injury	Reduced neutrophil recruitment, pulmonary edema, and inflammatory lung damage.	[27]
Candidiasis	p38γ/p38δ	Myeloid-specific knockout mice (LysM-Cre^+/−^-p38γ/δ^f/f^), experimental *C. albicans* infection; pharmacological inhibition (BIRB796)	Loss of p38γ/p38δ reshapes innate immunity and reduces infection-associated immunopathology, demonstrating TPL2-independent functions.	[18]
Collagen-induced arthritis	p38γ/p38δ	Mouse collagen-induced arthritis model (p38γ/δ^−/−^ mice, p38γ^−/−^, p38δ^−/−^ mice); human synovial macrophages and granulocytes	Deficiency of either kinase reduces disease severity, inflammatory cytokine production, and pathogenic anti-collagen antibody responses. Double knockout provides the strongest protection. p38 expression and phosphorylation are highly expressed in synovial tissue from rheumatoid arthritis patients.	[41,43,44]
Psoriasis/Chronic skin inflammation	p38δ (with p38α/p38β also implicated)	Human skin biopsies; cultured keratinocytes; differentiation studies	p38δ expression or activity is altered in keratinocyte differentiation and inflammatory signaling. However, human studies also implicate p38α and p38β; the available evidence therefore does not establish that the association is specific to p38δ.	[45,46]
Inflammation-associated skin carcinogenesis	p38γ/p38δ	p38γ/δ^−/−^, p38δ^−/−^, p38γ^−/−^ mice; DMBA/TPA skin carcinogenesis model	Combined deletion reduces skin inflammation and tumor promotion, linking chronic inflammation with epithelial tumorigenesis.	[47]
Colitis: Crohn’s disease, Ulcerative colitis	p38γ/p38δ	p38γ/δ^−/−^, p38δ^−/−^, p38γ^−/−^ mice; human plasma samples	p38γ/p38δ deficiency protects against DSS-induced acute colitis. p38γ and p38δ expression is altered in patients with colitis.	[8,42]
Colitis-associated colorectal cancer	p38γ/p38δ	p38γ/δ^−/−^, p38δ^−/−^, p38γ^−/−^ mice; AOM/DSS mouse model.	p38γ/p38δ deficiency reduces intestinal inflammation and protects against colitis-associated colon cancer by decreasing tumor number and size, an effect associated with reduced inflammatory cytokine production, altered immune-cell recruitment, and modulation of ERK1/2 signaling pathways.	[8,28]
Metabolic inflammation/Type 2 diabetes	p38δ	p38δ^−/−^ mice; high-fat diet; pancreatic β-cell studies	Improved glucose tolerance, enhanced insulin secretion, and protection from insulin resistance and oxidative β-cell dysfunction.	[48]
MASLD/MASH (metabolic dysfunction-associated steatohepatitis)	p38γ/p38δ	High-fat diet-fed LysM-Cre^+/−^-p38γ/δ^f/f^ mice; metabolic and histological analyses	p38γ/p38δ promote hepatic steatosis and metabolic inflammation, whereas genetic ablation reduces hepatic lipid accumulation, improves glucose homeostasis, and protects against diet-induced metabolic liver disease.	[49]
COPD/Airway mucus hypersecretion	p38δ	Human airway epithelial cells; siRNA; pharmacological inhibition; lung explants from COPD patients	IL-13 activates the CLCA1-p38δ pathway, inducing MUC5AC expression. Increased p38δ activation is observed in COPD lungs.	[50]

Summary of the current evidence linking p38γ and p38δ to inflammatory diseases. Evidence includes genetically modified mouse models, human patient samples, primary cells, transcriptomic analyses, and pharmacological inhibition studies. In most inflammatory settings, genetic or pharmacological inhibition of p38γ/p38δ attenuates cytokine production, inflammasome activation, tissue inflammation, or disease severity, highlighting these kinases as promising therapeutic targets.

## Therapeutic targeting: opportunities and challenges

Protection conferred by p38γ/p38δ deficiency in several experimental models supports further evaluation of these kinases as therapeutic targets in inflammatory disorders [2,10]. Their context-dependent roles in inflammatory and metabolic signaling, chronic inflammatory diseases, and inflammation-associated cancers provide a rationale for developing selective inhibitors and evaluating their preclinical efficacy. In addition, increased p38γ expression has been reported in several gastrointestinal and hepatobiliary malignancies, including colorectal and hepatocellular carcinomas, suggesting its involvement in these diseases. Further functional studies are required to establish its contribution to tumor development or progression and determine whether p38γ represents a valid therapeutic target [39,40].

Despite this strong biological rationale, the development of selective pharmacological inhibitors has proven challenging. The ATP-binding pocket is highly conserved among MAPKs, and the methionine gatekeeper residue that renders p38γ and p38δ insensitive to classical p38α/p38β inhibitors such as SB203580 has been difficult to exploit for selective drug design. Consequently, most available compounds, including BIRB796, display limited isoform selectivity and often inhibit additional kinases [17]. Nevertheless, several promising molecules have emerged, including the p38δ-selective Compound A, the inhibitors SU-002 and SU-005, AD80, CSH71, and the more recently developed p38δ inhibitor NuP-4 (Table 2). Although none has yet advanced to clinical development, these agents have provided important proof-of-concept that selective targeting of the alternative p38 MAPKs is feasible.

**Table 2:** Pharmacological tools targeting the alternative p38MAPKs p38γ and p38δ

Agent	Target	Mechanism	Key findings	Ref.
BIRB796 (Doramapimod)	p38α/β/γ/δ	Type II (DFG-out) inhibitor	Inhibits all p38 isoforms, although with lower potency toward p38γ/δ; widely used to study alternative p38 MAPKs.	[17]
Compound A	p38δ	ATP-competitive inhibitor	First selective p38δ inhibitor validated *in vivo*; reduced PKD1 phosphorylation, neutrophil recruitment, and LPS-induced acute lung injury.	[27]
SU-002/SU-005	p38γ/δ	ATP-competitive inhibitor	First proof-of-concept compounds exploiting the Met gatekeeper residue for preferential p38γ/δ inhibition.	[51]
AD80	p38γ/δ (multikinase)	ATP-competitive inhibitor	Potent inhibitor of p38γ/δ activity; widely used in mechanistic and cancer studies.	[52]
CSH71	p38γ	Lipid-binding-site (LBS) inhibitor; also engages the ATP pocket	Improved selectivity toward p38γ; useful chemical probe for functional studies.	[53]
NuP-3	p38δ/p38α	ATP-competitive inhibitor	Suppress IL-13-driven p38δ signalling, mucus production, and airway inflammation in preclinical models.	[54]
NuP-4	p38δ	ATP-competitive inhibitor	First highly selective p38δ inhibitor; suppresses cytokine-driven immune activation, epithelial stem-cell reprogramming, and pathological tissue remodeling.	[55]
F7 (PIK75)	p38γ (multikinase)	ATP-competitive inhibitor	Selectively kills malignant CTCL cells, inhibits p38γ activity and DLGH1 phosphorylation, and reduces tumor growth in CTCL xenograft models. Identified p38γ as a therapeutic vulnerability in CTCL.	[56]
WFYH	p38δ	Peptide-based ATP-pocket inhibitor	Selectively inhibits p38δ over p38α; developed from structure-based design and proposed as a therapeutic approach for HNSCC.	[57]
p38δ PROTACs (SJF series)	p38δ	Targeted protein degradation	First selective degradation of p38δ, demonstrating isoform-selective PROTAC technology.	[58]

Summary of the main pharmacological tools developed to target p38γ and p38δ MAPKs, including ATP-competitive inhibitors, dual p38α/p38δ inhibitors and targeted protein degraders (PROTACs). The table summarizes their selectivity, mechanism of action, principal applications and current development status.

In this context, pirfenidone, a clinically approved antifibrotic agent used to treat idiopathic pulmonary fibrosis [59], has been used experimentally to inhibit p38γ signaling in inflammatory and cancer models [28,60]. However, the specificity and potency of pirfenidone toward p38γ remain uncertain. Although pirfenidone has been reported to inhibit p38γ at concentrations above 100 μM [61,56], it showed little or no inhibitory activity at 10 μM in kinase profiling performed by the MRC Protein Phosphorylation and Ubiquitylation Unit, with 96% residual p38γ activity [62]. Moreover, pirfenidone exerts broad antifibrotic and anti-inflammatory effects through multiple cytokines and signaling pathways, consistent with a pleiotropic rather than isoform-selective mechanism of action. Accordingly, some of the effects observed in these models may reflect broader anti-inflammatory actions rather than selective p38γ inhibition. Thus, pirfenidone does not clinically validate p38γ as a molecular target, highlighting the need for more potent and isoform-selective inhibitors.

Beyond these limitations in inhibitor selectivity and potency, some critical functions of p38γ and p38δ are independent of kinase activity. Notably, both proteins contribute to the stabilization of TPL2, a function that relies on the presence of the protein rather than its catalytic activity. As a result, ATP-competitive inhibitors may not fully reproduce the phenotypes observed in genetic knockout models. This concept is illustrated by the p38γ/δ^KIKO^ mouse, in which the kinases are catalytically inactive but still expressed. The protection observed in these mice against septic shock and candidiasis demonstrates that kinase inhibition can recapitulate part of the beneficial effects of p38γ/p38δ deficiency, whereas differences between knockin and complete knockout models indicate that some protective functions require elimination of the protein itself [22].

These observations suggest that targeted protein degradation may represent a particularly attractive therapeutic strategy, as it could simultaneously eliminate both the catalytic and scaffolding functions of p38γ and p38δ. The recent development of p38α/p38δ-directed PROTACs provides encouraging evidence that more effective and selective pharmacological approaches may be achievable [58]. Overall, the development of potent isoform-selective inhibitors and degraders will be crucial for fully defining and exploiting the therapeutic potential of p38γ and p38δ.

## Conclusions and future directions

Over the past decade, p38γ and p38δ have emerged as important regulators of inflammation, with isoform-specific, overlapping, and potentially compensatory functions. Although mice lacking p38γ, p38δ, or both isoforms are viable and show no major overt abnormalities under standard laboratory conditions, multiple phenotypes emerge following inflammatory, infectious, metabolic, or oncogenic challenges. This supports context-dependent functions that may be masked by compensatory mechanisms under basal conditions. Evidence from genetic models further demonstrates their contribution to a broad spectrum of inflammatory and inflammation-associated diseases, including colitis-associated colorectal cancer and metabolic disorders.

Future research should combine isoform-selective inhibitors and degraders with inducible, cell-type-specific loss- and gain-of-function models to define the relative contributions of p38γ and p38δ in different cellular and disease contexts. Parallel analysis of all four p38 isoforms will help identify functional compensation and signaling redistribution, whereas direct activity reporters and studies in patient-derived material will distinguish expression-based associations from functional dependencies. Establishing p38γ/p38δ expression and activity signatures in patient samples will also be essential to identify responsive patient populations and prioritize therapeutic indications. Together, these advances should provide the mechanistic and translational framework required to develop selective therapies targeting p38γ and p38δ while anticipating potential effects on host defense and tissue homeostasis.

## Perspectives

- p38γ and p38δ are important regulators of innate and adaptive immunity whose contributions may be isoform-specific, overlapping, or compensatory depending on the cellular and inflammatory context. Their genetic ablation protects mice from a broad spectrum of inflammatory diseases, highlighting these p38MAPKs as promising therapeutic targets.
- p38γ and p38δ regulate inflammatory responses through both kinase-dependent and kinase-independent mechanisms and signal through non-canonical pathways that control cytokine production, immune-cell activation, and inflammatory tissue damage.
- The development of isoform-selective inhibitors and degraders, together with a better understanding of the catalytic and scaffolding functions of each isoform and their relevance in human disease, will be crucial to establish their therapeutic potential.

## Open Access

Open access for this article was enabled by the participation of the Centro Nacional de Biotecnología in an all-inclusive Read & Publish agreement with Portland Press and the Biochemical Society under an agreement with the CSIC.

## Abbreviations

ABIN2: A20-binding inhibitor of NF-κB 2
ATF1: activating transcription factor 1
CARM1: coactivator-associated arginine methyltransferase 1
CREB: cAMP-response element-binding protein
eEF2K: eukaryotic elongation factor 2 kinase
ERK: extracellular signal-regulated kinase
hDlg: human discs large protein (DLG1)
IL: interleukin
iNOS: inducible nitric oxide synthase
JAK: Janus kinase
KRAS: Kirsten rat sarcoma viral oncogene homolog
LPS: lipopolysaccharide
MAP3K: MAP kinase kinase kinase
MAPK: mitogen-activated protein kinase
MASLD: metabolic dysfunction-associated steatotic liver disease
MEF2D: myocyte enhancer factor-2D
MKK or MAP2K: MAP kinase kinase
MSK1: mitogen- and stress-activated protein kinase 1
NAFLD: non-alcoholic fatty liver disease
NF-κB: nuclear factor κB
p62/SQSTM1: sequestosome
PDZ: PSD-95/Dlg/ZO-1 domain
PI3K: phosphoinositide 3-kinase
PKD1: protein kinase D1
PTEN: phosphatase and tensin homolog
ROS: reactive oxygen species
SAPK: stress-activated protein kinase
STAT: signal transducer and activator of transcription
TLR: Toll-like receptor
TNFα: tumor necrosis factor-α
TPL2: tumor progression locus 2
Wnt: Wingless/Integrated signaling pathway
